# Swedish experience-based value sets for EQ-5D health states

**DOI:** 10.1007/s11136-013-0496-4

**Published:** 2013-08-22

**Authors:** Kristina Burström, Sun Sun, Ulf-G Gerdtham, Martin Henriksson, Magnus Johannesson, Lars-Åke Levin, Niklas Zethraeus

**Affiliations:** 1 Department of Learning, Informatics, Management and Ethics, Medical Management Centre, Karolinska Institutet, Tomtebodavägen 18A, 171 77 Stockholm, Sweden; 2 Department of Public Health Sciences, Equity and Health Policy Research Group, Karolinska Institutet, Tomtebodavägen 18A, 171 77 Stockholm, Sweden; 3 Stockholm County Council, Health Care Services, Tomtebodavägen 18A, 171 77 Stockholm, Sweden; 4 Department of Economics, Lund University, P.O. Box 7082, 220 07 Lund, Sweden; 5 Health Economics and Management, Institute of Economic Research, Lund University, P.O. Box 7082, 220 07 Lund, Sweden; 6 Centre for Primary Health Care Research, Lund University, P.O. Box 7082, 220 07 Lund, Sweden; 7 Department of Health Economics, AstraZeneca Nordic, 151 85 Södertälje, Sweden; 8 Department of Economics, Stockholm School of Economics, P.O. Box 6501, 113 83 Stockholm, Sweden; 9 Department of Medical and Health Sciences, Center for Medical Technology Assessment, Linköping University, 581 83 Linköping, Sweden; 10 The Dental and Pharmaceutical Benefits Agency, P.O. Box 225 20, 104 22 Stockholm, Sweden

**Keywords:** EQ-5D, Experience-based value set, General population, Self-rated health, Time trade off, Visual analogue scale

## Abstract

**Purpose:**

To estimate Swedish experience-based value sets for EQ-5D health states using general population health survey data.

**Methods:**

Approximately 45,000 individuals valued their current health status by means of time trade off (TTO) and visual analogue scale (VAS) methods and answered the EQ-5D questionnaire, making it possible to model the association between the experience-based TTO and VAS values and the EQ-5D dimensions and severity levels. The association between TTO and VAS values and the different severity levels of respondents’ answers on a self-rated health (SRH) question was assessed.

**Results:**

Almost all dimensions (except usual activity) and severity levels had less impact on TTO valuations compared with the UK study based on hypothetical values. Anxiety/depression had the greatest impact on both TTO and VAS values. TTO and VAS values were consistently related to SRH. The inclusion of age, sex, education and socioeconomic group affected the main effect coefficients and the explanatory power modestly.

**Conclusions:**

A value set for EQ-5D health states based on Swedish valuations has been lacking. Several authors have recently advocated the normative standpoint of using experience-based values. Guidelines of economic evaluation for reimbursement decisions in Sweden recommend the use of experience-based values for QALY calculations. Our results that anxiety/depression had the greatest impact on both TTO and VAS values underline the importance of mental health for individuals’ overall HRQoL. Using population surveys is in line with recent thinking on valuing health states and could reduce some of the focusing effects potentially appearing in hypothetical valuation studies.

**Electronic supplementary material:**

The online version of this article (doi:10.1007/s11136-013-0496-4) contains supplementary material, which is available to authorized users.

## Introduction

EQ-5D is a generic health-related quality of life (HRQoL) instrument from which a single-index value of the respondent’s health status can be derived, based on a health profile consisting of five dimensions with three severity levels [1]. EQ-5D is commonly used to estimate the quality-of-life component in quality-adjusted life years (QALYs) in the economic evaluation of health technologies, and also as a health care performance indicator and in the measurement of population health in surveys [2–6]. In determining values for the 243 health states defined by the EQ-5D, referred to as a value set, methods of valuation as well as the source of the valuations must be considered [7–10]. Although EQ-5D has been widely adopted in Sweden for economic evaluation, no value set based on a Swedish population has yet been developed.

A value set can be obtained using different methods for the valuation of health states: the time trade off (TTO), the standard gamble (SG), the rating scale (RS) and the discrete choice (DC) method [4, 11]. A central question is whether the valuations should be based on preferences from individuals who are actually in the health state, i.e., experience-based values, or from individuals to whom the health states are described, i.e., hypothetical values [8, 12–22]. The former is often denoted patient or individual values, and the latter social values (a sample of the general public has valued imagined health states). Experience-based values refer to the value of the individual’s currently experienced health state. However, in the valuation process, also imagined states are involved: worst and best, full health and dead.

A country-specific value set for EQ-5D health states was first generated in the UK [23] based on hypothetical values derived from a sample of the general population using the TTO method [9]. Country-specific value sets based on VAS data for hypothetical health states [9], a regional value set based on aggregated VAS data from six European countries [24] and a TTO value set from a Hispanic population in the US [25] have also been generated. Cross-country value set comparison studies suggest that there might be substantial differences in values across countries [9, 26–32].

Hypothetical values have been supported by the arguments that health policies and interventions affect us all (as tax payers and potential patients) and that the adaptation to a health state should not be reflected in valuations [33]. In contrast, advantages of using experience-based values based on preferences from the best informed [12, 14–16, 34–36] imply that adaptation will be reflected in the valuations [37–39]. Experience-based values tend to be higher than hypothetical values [8, 22, 39].

An experience-based VAS value set for EQ-5D has been developed for the German population [35]. Experience-based TTO values for EQ-5D health states have also been investigated [16] as well as experience-based VAS values [34, 36].

The National Institute for Health and Clinical Excellence (NICE) in England and Wales recommends using the UK EQ-5D ‘social tariff’ based on hypothetical values for QALY weightings [40]. In Sweden, the Dental and Pharmaceutical Benefits Agency (TLV) states that QALY weightings can be based either on direct or indirect measurements (‘where a health classification system such as EQ-5D is linked to QALY weightings’) and that ‘QALY weightings based on appraisals of persons in the health condition in question are preferred before weightings calculated from an average of a populations estimating a condition depicted for it (e.g., the ‘social tariff’ from EQ-5D)’ [41]. Thus, TLV prefers experience-based rather than hypothetical values [41].

This study attempted to estimate Swedish experience-based value sets for EQ-5D health states using general population health survey data. In the surveys, respondents valued their current health status by means of the TTO and VAS methods and answered the EQ-5D questionnaire, making it possible to model the association between the experience-based TTO and VAS values and the dimensions and severity levels of the EQ-5D instrument. For validation purposes, the association between TTO and VAS values and different severity levels of respondents’ answers on a self-rated health (SRH) question was investigated. Supplementary detail on data and variables as well as results is found in Online Resource (11136_2013_496_MOESM1_ESM.pdf).

## Data and variables

### Material/study population

In this study, we use large cross-sectional population-based health surveys from two areas in Sweden representing one-third of the Swedish population. The surveys (the Public Health Survey in Scania Region 2004 and the Public Health Survey in Stockholm County 2006) included the same questions. The analysis was carried out on a pooled data set.

In Sweden, a majority of the population live in urban areas. The socio-demographic composition of Scania Region resembles to a greater extent Sweden as a whole than does Stockholm County (Online Resource Table S1), where a smaller proportion live outside urban areas, and the mean age is lower; the educational and income levels are higher.

Self-administered postal questionnaires, with three reminders, were sent out to representative samples stratified by sex and geographic area. The EQ-5D self-report descriptive system, a visual analogue scale (EQ VAS), a time trade off (TTO) question and a SRH question were included in the surveys along with questions on living habits and conditions.

Data consisted of responses from 51,254 individuals, aged 18–80 years. See Online Resource for details.

The anonymised data are based on information from individuals who agreed to participate (informed consent), and respondents cannot be traced. Ethical approval was granted by the Regional Ethical Review Board, Stockholm (Dnr: 2011/582-31/5).

### The EQ-5D descriptive system

With the EQ-5D descriptive system, respondents classify their own health status into five dimensions: mobility; self-care; usual activities; pain/discomfort; anxiety/depression, within three levels of severity: no problems; moderate problems; severe problems, defining 243 health states (profiles) [1].

### The EQ VAS

On the EQ VAS, respondents rate their own overall health status on a vertical VAS (100 = best imaginable health; 0 = worst imaginable health).

### The TTO question

The TTO question consisted of a horizontal line, representing 0–10 years, where every year was marked and labelled 0, 1, 2, …, 10 years [42]. Every half year was marked, but not labelled. A similar TTO question has been employed in other studies [13, 16, 43, 44].

### The SRH question

The SRH question was phrased *‘In your opinion, how is your health status? Is it very good, good, fair, bad, very bad’?*

## Methods

We performed regression analysis on the individual data of all respondents with TTO and VAS values as the dependent variables. The variables and the definition of models are presented in Table 1. See Online Resource for analyses stratified by survey and test for parameter homogeneity across surveys.

**Table 1:** Definition of variables and models

Variable	Definition
MO2	1 if mobility at level 2; 0 otherwise
MO3	1 if mobility at level 3; 0 otherwise
MO23	1 if mobility at level 2 or 3; 0 otherwise
SC2	1 if self-care at level 2; 0 otherwise
SC3	1 if self-care at level 3; 0 otherwise
SC23	1 if self-care at level 2 or 3; 0 otherwise
UA2	1 if usual activities at level 2; 0 otherwise
UA3	1 if usual activities at level 3; 0 otherwise
PD2	1 if pain/discomfort at level 2; 0 otherwise
PD3	1 if pain/discomfort at level 3; 0 otherwise
AD2	1 if anxiety/depression at level 2; 0 otherwise
AD3	1 if anxiety/depression at level 3; 0 otherwise
N3	1 if any dimension at level 3; 0 otherwise
SRH2	1 if SRH is good health; 0 otherwise
SRH3	1 if SRH is fair health; 0 otherwise
SRH4	1 if SRH is bad health; 0 otherwise
SRH5	1 if SRH is very bad; 0 otherwise

TTO models on EQ-5D	*f* (*x*)
Model 1	*f* (MO2 MO3 SC2 SC3 UA2 UA3 PD2 PD3 AD2 AD3)
Model 2	*f* (MO2 MO3 SC2 SC3 UA2 UA3 PD2 PD3 AD2 AD3 N3)
Model 3	*f* (MO2 MO3 SC23 UA2 UA3 PD2 PD3 AD2 AD3)
Model 4	*f* (MO2 MO3 SC23 UA2 UA3 PD2 PD3 AD2 AD3 N3)
Model 5	*f* (MO2 MO3 SC23 UA2 UA3 PD2 PD3 AD2 AD3 N3 age)
Model 6	*f* (MO2 MO3 SC23 UA2 UA3 PD2 PD3 AD2 AD3 N3 age sex)
Model 7	*f* (MO2 MO3 SC23 UA2 UA3 PD2 PD3 AD2 AD3 N3 age sex educational level socioeconomic group)

VAS models on EQ-5D	*f* (*x*)
Model 1	*f* (MO2 MO3 SC2 SC3 UA2 UA3 PD2 PD3 AD2 AD3)
Model 2	*f* (MO2 MO3 SC2 SC3 UA2 UA3 PD2 PD3 AD2 AD3 N3)
Model 3	*f* (MO23 SC23 UA2 UA3 PD2 PD3 AD2 AD3)
Model 4	*f* (MO23 SC23 UA2 UA3 PD2 PD3 AD2 AD3 N3)
Model 5	*f* (MO23 SC23 UA2 UA3 PD2 PD3 AD2 AD3 N3 age)
Model 6	*f* (MO23 SC23 UA2 UA3 PD2 PD3 AD2 AD3 N3 age sex)
Model 7	*f* (MO23 SC23 UA2 UA3 PD2 PD3 AD2 AD3 N3 age sex educational level socioeconomic group)

TTO and VAS models on SRH	*f* (*x*)
Model 1	*f* (SRH2 SRH3 SRH4 SRH5)
Model 2	*f* (SRH2 SRH3 SRH4 SRH5 age)
Model 3	*f* (SRH2 SRH3 SRH4 SRH5 age sex)
Model 4	*f* (SRH2 SRH3 SRH4 SRH5 age sex educational level socioeconomic group)

*TTO* time trade off, *VAS* visual analogue scale, *SRH* self-rated health

The individual TTO value was calculated by dividing the response to the TTO question by 10. The trade off was between x years in full health versus 10 years in the respondent’s current health state; shorter time implied worse health state. The individual VAS value was given by the numeric value on the VAS between 0 and 100. The VAS values were not rescaled to be anchored on dead and full health and could hence not directly be used in QALY calculations.

A set of ten dummy variables was representing the main effect within each of the five EQ-5D dimensions (Model 1) [23]. The dummy variables for level 2 represents the main effect of movement from level 1 (no problems) to level 2 (moderate problems), and the dummy variables for level 3 represents the main effect of movement from level 1 (no problems) to level 3 (severe problems) (Table 1).

Interaction variables were tested: first order interaction effects between the five EQ-5D dimensions; if levels 2 or 3 in any of the dimensions (N2 and N3, respectively); the number and the square of the number of dimensions on levels 2 or 3; whether there are two or more, three or more, four or more, or five dimensions on levels 2 or 3.

We expected consistent ordering between the levels, i.e., that all coefficients should have a negative sign and that the coefficient for severe problems should be greater in absolute terms than the coefficients for moderate problems. However, in the TTO analyses for the self-care dimension, the coefficient for severe problems was smaller than the coefficient for moderate problems, and this could not be handled by entering interaction or other nonlinear variables in any of the models. Therefore, we merged levels 2 and 3 into one category and entered a dummy variable (SC23) representing any move from level 1 (no problems) in the self-care dimension. In the VAS analyses, a similar inconsistency was observed for the self-care and mobility dimensions, and additional dummy variables (SC23 and MO23) were entered. None of the interaction coefficients were significant, except the N3 variable, and were not entered in the final model.

We estimated ordinary least squares (OLS) regressions with the ten dummy variables for the dimensions (Model 1) and with the N3 variable (Model 2). We merged levels 2 and 3 for self-care or mobility (Model 3) and included the N3 variable (Model 4), respectively, in the TTO and VAS regressions.

Statistical tests were employed to evaluate the models’ goodness-of-fit: the estimated values predicted by the models were compared with the observed values by calculating Spearman’s correlation coefficients and the mean absolute difference (MAD). The higher correlations, the better the model fit and the smaller MAD, the better the model fit.

The final choice of model specification (Model 4) for the estimation of TTO and VAS value sets was based on the following criteria: the ordinal nature of the severity levels within each dimension should be reflected (consistency); how well the model explains the differences between estimated and observed health state values (goodness-of-fit); the simplicity of the model (parsimony); and that non-experts can understand the modelling (transparency) [45].

To estimate the robustness of the final model, a split sample test was employed, where the total sample was randomly divided into two groups of equal size [23]. Estimations from one group were used to predict the values in the other group.

In addition, we investigated the effect of age (Model 5), sex (Model 6), education and socioeconomic group (Model 7) on valuation of health states (Table 1) (see Online Resource for classification).

Furthermore, we explored the effect of SRH on TTO and VAS values, respectively (Table 1). Dummy variables were created representing the severity levels (reference group very good health) (Model 1). The dummy variables represent the effect of movement from very good health to good, fair, bad and very bad health, respectively. We estimated the regression models with and without the above-described socio-demographic dummy variables (Models 2–4).

Since there were indications of heteroscedasticity, robust estimates were employed [46]. A 5 % significance level was used. All analyses were carried out in SAS Version 9.2 [47].

## Results

Of the 243 possible health states of the EQ-5D descriptive system, 148 health states were reported and valued. Socio-demographic characteristics and self-reported health measures for the pooled data are presented in Table 2. See Online Resource Table S2 for characteristics by survey.

**Table 2:** Characteristics of the respondents, pooled data

Variable	18–80 years	
	(*n* = 49,169)	
	%	*n*
Women	56.3	27,700
Mean age (years)	46.2	49,169
Age group		
18–24 years	9.1	4,483
25–34 years	16.8	8,239
35–44 years	20.9	10,295
45–54 years	19.9	9,804
55–64 years	22.6	11,108
65–74 years	7.5	3,692
75–80 years	3.2	1,548
Educational level		
Low	17.1	8,414
Medium	42.1	20,703
High	37.0	18,172
Missing	3.8	1,880
Socioeconomic group		
Unskilled manual	17.9	8,788
Skilled manual	12.1	5,949
Lower non-manual	10.9	5,362
Intermediate non-manual	18.7	9,186
Higher non-manual	13.7	6,751
Self-employed and farmers	4.0	1,989
Other	22.7	11,144
Less than good SRH	27.7	13,593
Mobility		
Moderate problems (level 2)	9.8	4,840
Severe problems (level 3)	0.1	50
Self-care		
Moderate problems (level 2)	1.2	600
Severe problems (level 3)	0.4	198
Usual activities		
Moderate problems (level 2)	7.7	3,785
Severe problems (level 3)	1.1	536
Pain/discomfort		
Moderate problems (level 2)	45.1	22,185
Severe problems (level 3)	4.1	2,038
Anxiety/depression		
Moderate problems (level 2)	30.8	15,126
Severe problems (level 3)	2.7	1,322
Problems in at least one EQ-5D dimension	60.2	29,618
Problems on level 3	6.7	3,287
TTO (mean)	0.91	45,477
EQ VAS (mean)	79.5	41,761

*TTO* time trade off, *VAS* visual analogue scale, *SRH* self-rated health

### Regression analysis on TTO values for EQ-5D dimensions

The results of the regression analysis on individual TTO values for EQ-5D dimensions are presented in Table 3.

**Table 3:** Regression analysis on TTO values, EQ-5D dimensions

Variable	Model 1		Model 2		Model 3		Model 4		Model 5		Model 6		Model 7	
	Estimate	*p* value	Estimate	*p* value	Estimate	*p* value	Estimate	*p* value	Estimate	*p* value	Estimate	*p* value	Estimate	*p* value
Intercept	0.9692	<0.0001	0.9693	<0.0001	0.9693	<0.0001	0.9694	<0.0001	0.9606	<0.0001	0.9527	<0.0001	0.9480	<0.0001
*Mobility*														
Level 2	−0.0665	<0.0001	−0.0660	<0.0001	−0.0668	<0.0001	−0.0666	<0.0001	−0.0638	<0.0001	−0.0634	<0.0001	−0.0623	<0.0001
Level 3	−0.1464	<0.0001	−0.1500	0.0006	−0.1298	0.0025	−0.1247	0.0032	−0.1234	0.0038	−0.1218	0.0043	−0.1217	0.0043
*Self*-*care*														
Level 2	−0.0490	<0.0001	−0.0477	0.0002	–	–	–	–	–	–	–	–	–	–
Level 3	0.0068	0.7308	0.0445	0.0481	–	–	–	–	–	–	–	–	–	–
Level 2 and 3	–	–	–	–	−0.0350	0.0015	−0.0276	0.0161	−0.0254	0.0273	−0.0242	0.0350	−0.0233	0.0425
*Usual activities*														
Level 2	−0.1014	<0.0001	−0.0994	<0.0001	−0.1022	<0.0001	−0.1012	<0.0001	−0.1031	<0.0001	−0.1036	<0.0001	−0.1029	<0.0001
Level 3	−0.1483	<0.0001	−0.1331	<0.0001	−0.1469	<0.0001	−0.1355	<0.0001	−0.1364	<0.0001	−0.1363	<0.0001	−0.1359	<0.0001
*Pain/discomfort*														
Level 2	−0.0347	<0.0001	−0.0346	<0.0001	−0.0347	<0.0001	−0.0345	<0.0001	−0.0348	<0.0001	−0.0355	<0.0001	−0.0337	<0.0001
Level 3	−0.1236	<0.0001	−0.0759	<0.0001	−0.1242	<0.0001	−0.0904	<0.0001	−0.0926	<0.0001	−0.0935	<0.0001	−0.0911	<0.0001
*Anxiety/depression*														
Level 2	−0.0555	<0.0001	−0.0550	<0.0001	−0.0555	<0.0001	−0.0552	<0.0001	−0.0551	<0.0001	−0.0566	<0.0001	−0.0562	<0.0001
Level 3	−0.2393	<0.0001	−0.1948	<0.0001	−0.2393	<0.0001	−0.2077	<0.0001	−0.2084	<0.0001	−0.2092	<0.0001	−0.2083	<0.0001
*N3*	–	–	−0.0607	<0.0001	–	–	−0.0433	0.0017	−0.0427	0.0020	−0.0433	0.0017	−0.0429	0.0018
*Age group* ^*a*^														
25–34	–	–	–	–	–	–	–	–	0.0097	0.0010	0.0096	0.0012	0.0030	0.3305
35–44	–	–	–	–	–	–	–	–	0.0080	0.0063	0.0084	0.0040	0.0008	0.7880
45–54	–	–	–	–	–	–	–	–	0.0135	<0.0001	0.0144	<0.0001	0.0071	0.0217
55–64	–	–	–	–	–	–	–	–	0.0158	<0.0001	0.0170	<0.0001	0.0096	0.0013
65–74	–	–	–	–	–	–	–	–	0.0038	0.2996	0.0051	0.1588	0.0007	0.8557
75–80	–	–	–	–	–	–	–	–	−0.0413	<0.0001	−0.0397	<0.0001	−0.0329	<0.0001
*Sex* ^*b*^	–	–	–	–	–	–	–	–	–	–	0.0144	<0.0001	0.0137	<0.0001
*Educational level* ^*c*^														
Medium	–	–	–	–	–	–	–	–	–	–	–	–	0.0020	0.3889
High	–	–	–	–	–	–	–	–	–	–	–	–	0.0045	0.0825
Missing	–	–	–	–	–	–	–	–	–	–	–	–	−0.0115	0.0818
*Socioeconomic group* ^*d*^														
Skilled manual	–	–	–	–	–	–	–	–	–	–	–	–	0.0065	0.0229
Lower non-manual	–	–	–	–	–	–	–	–	–	–	–	–	0.0120	<0.0001
Intermed non-manual	–	–	–	–	–	–	–	–	–	–	–	–	0.0132	<0.0001
Higher non-manual	–	–	–	–	–	–	–	–	–	–	–	–	0.0161	<0.0001
Self-employed	–	–	–	–	–	–	–	–	–	–	–	–	0.0166	<0.0001
Other	–	–	–	–	–	–	–	–	–	–	–	–	0.0010	0.6975
Observations	45.477		45.477		45.477		45.477		45.477		45.477		45.477	
Adjusted *R* ^2^	0.2385		0.2393		0.2383		0.2387		0.2415		0.2431		0.2446	
*F* statistics^e, f^	*F*_(1,41185)_ = 108.39*		*F*_(1,41184)_ = 106.78*		*F*_(1,41186)_ = 106.95*		*F*_(1,41185)_ = 105.43*		*F*_(1,41185)_ = 105.43*		*F*_(1,41185)_ = 105.43*		*F*_(1,41185)_ = 105.43*	
*F* statistics^e, g^									*F*_(19,41197)_ = 4.20*		*F*_(10,41175)_ = 12.42*		*F*_(28,41157)_ = 6.72*	
*F* statistics^e, h^	*F*_(10,41175)_ = 6.21*		*F*_(11,41173)_ = 5.54*		*F*_(9,41177)_ = 6.72*		*F*_(10,41175)_ = 5.88*		*F*_(29,41167)_ = 5.94*		*F*_(10,41165)_ = 5.90*		*F*_(10,41147)_ = 6.11*	

*TTO* time trade off

* *p* = 0.001

^a^Reference group: 18–24 years

^b^Reference group: men

^c^Reference group: low educational level

^d^Reference group: unskilled manual workers

^e^Age restricted to 18–64 years

^f^ *F* test of equal intercepts in the two surveys

^g^ *F* test of equal parameters of all non-dimensional regressors in the two surveys

^h^ *F* test of equal parameters of all dimensional regressors in the two surveys

Model 1 includes the main effect within each of the five dimensions with dummy variables entered for moderate and severe levels. The TTO results were consistent that the values were lower, the more severe the health state, except for self-care where the coefficient for level 3 was not lower compared to level 2. The N3 variable had a negative sign and was significant (Model 2). Entering the SC23 variable (merged levels 2 and 3 for self-care) resulted in all coefficients for all dimensions becoming statistically significant irrespective of the exclusion (Model 3) or inclusion (Model 4) of the N3 variable.

For health states with ten or more observations, the Spearman’s correlation coefficients were greater and the MAD smaller, compared with states with five or more observations (Table 4). For health states with five or more observations, the correlation coefficient was greater and the MAD was smaller in Model 4 compared to Model 3. For health states with ten or more observations, the correlation coefficient was greater and the MAD was smaller in Model 3. However, the differences between all models were small. The adjusted *R* ^2^ was similar for all four models (around 0.24) (Table 3).

**Table 4:** Correlation and mean absolute difference (MAD), TTO values, EQ-5D

Number of health states	TTO							
	Model 1		Model 2		Model 3		Model 4	
	Correlation	MAD	Correlation	MAD	Correlation	MAD	Correlation	MAD
*n* ≥ 5	0.833	0.0552	0.849	0.0506	0.824	0.0560	0.830	0.0539
*n* ≥ 10	0.936	0.0389	0.934	0.0385	0.933	0.0404	0.928	0.0408

*TTO* time trade off

**Fig. 1:**
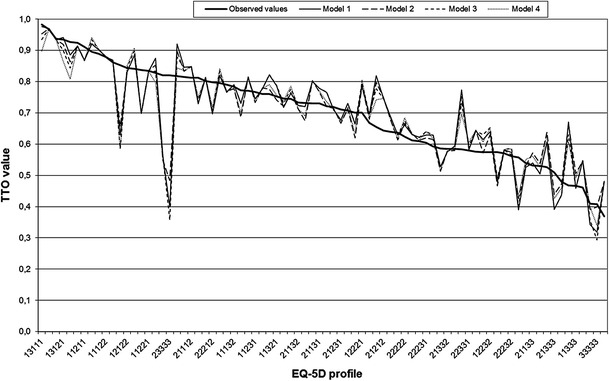
Estimated TTO values predicted by different OLS models compared to the observed mean TTO values for health states with five or more observations (*n* = 80)

Figure 1 shows the estimated TTO values predicted by the different OLS models compared to the observed mean TTO values for health states with five or more observations.

The consistency criteria together with the goodness-of-fit analyses and the fact that the N3 variable was significant resulted in Model 4 being the best-fitting model for the data. Severe problems with anxiety/depression had the greatest effect (0.208), followed by severe problems with usual activities (0.136), mobility (0.125) and pain/discomfort (0.090) (Table 3). For moderate problems, the greatest coefficient was seen for usual activities (0.101) followed by mobility (0.067), anxiety/depression (0.055) and pain/discomfort (0.035). The merged coefficient for self-care (0.028) was interpreted as any move away from no problems. The difference between the predicted and the observed mean values exceeded 0.1 for 15 % of the health states with five or more observations.

The coefficients for age were significantly positive for nearly all age groups and negative for the oldest (Model 5). The coefficient for sex was significant with higher TTO values for women (Model 6), while the coefficients for educational level were not significant (Model 7). The coefficients for socioeconomic groups were positive and significant. Including age, sex, education and socioeconomic group affected the main effect coefficients modestly and increased the adjusted *R* ^2^ from 0.239 to 0.245.

A Swedish TTO value set, based on Model 4, for the 243 EQ-5D health states, is presented in Online Resource Table S3.

### Regression analysis on VAS values for EQ-5D dimensions

Corresponding results of the regression analysis on individual VAS values for EQ-5D dimensions and model comparison are presented in Online Resource Tables S4–S5 and Figure S1.

A Swedish VAS value set, based on Model 4, for the 243 health states, is presented in Online Resource Table S3.

Comparison of TTO and VAS values for Models 2 and 4 is presented in Online Resource Table S6 (pooled data) and Table S7 (by survey).

### Regression analysis on TTO values and VAS values for SRH

Corresponding results of the regression analysis on individual TTO and VAS values for SRH levels are presented in Online Resource Table S8.

The TTO and VAS values for the different severity levels for SRH are presented in Online Resource Table S9.

Comparison of TTO and VAS values for SRH levels is presented in Online Resource Table S10 (pooled data) and Table S11 (by survey).

## Discussion

Although tentative valuation studies have been performed previously in Sweden [13, 48], this is the first attempt to estimate a Swedish value set for EQ-5D health states. In two large cross-sectional population-based surveys, individuals described their current health status in the EQ-5D descriptive system and valued their health status using TTO and VAS. In line with recent studies [16, 34, 35], statistical modelling was used to model the association between the experience-based values and the dimensions and severity levels of the EQ-5D descriptive system. The preferred OLS models included an N3 variable and discriminated consistently between severity levels in the five dimensions except for self-care for both TTO and VAS, and mobility for VAS. Other studies have also encountered inconsistencies with coefficients having the wrong sign or being non-significant; several studies also appeared to encounter similar issues with self-care [35, 49–51], possibly due to the low prevalence of any problems on the self-care dimension. Age could also be a factor as problems with self-care are more prevalent among older respondents, who may be more prone to misunderstand the valuation task [52].

To handle the inconsistencies, we merged levels 2 and 3 resulting in a significant coefficient for self-care in the TTO regression. In the VAS regression, the re-specification resulted in a significant coefficient for mobility, but not for self-care (although this coefficient was kept as it had the correct sign). With this approach, the N3 variable still distinguishes between moderate and severe problems within self-care and mobility for health states where no other dimension is at the severe level as this coefficient is applied only once if any dimension is at the severe level. Inconsistent coefficients have sometimes been omitted altogether [35, 50, 52], implying that health states are assigned the same value in the value set, whereas other have kept insignificant (but logically plausible) coefficients [23, 51]. As inconsistencies occur in both experience-based studies and studies based on hypothetical values, other explanations than sources of valuations are likely. The MAD for our TTO and VAS models were smaller compared with other studies [53].

Although caution is warranted in comparing different studies [32], our TTO value set shows a general trend towards higher values compared with the UK TTO value set [23]. Almost all dimensions and severity levels in our study have less impact on TTO valuations (except usual activity) compared with the UK study. In particular, the differences appear more pronounced for severe health states as indicated by the much smaller coefficients for level 3 and N3 in our study; a similar trend was observed when comparing our results to Danish hypothetical TTO values [53]. Previous studies have shown that experience-based values tend to be higher than hypothetical values, in particular for severe health states [13, 22, 36, 39, 54, 55]. The relative importance of the health dimensions also appears to differ between experience-based and hypothetical values; problems in the mood dimension seem to be valued worse when values are experience-based [8, 12, 14].

Our study sample has strengths and weaknesses. The size of our study sample provides a strong foundation for the statistical modelling. Approximately 45,000 individuals provided valuations of about 60 % of the EQ-5D health states, and 80 of the health states were valued by five or more individuals. A potential limitation is that two cross-sectional population-based health surveys from different areas of Sweden were used in the absence of a national sample of Sweden. However, our large sample represents one-third of the Swedish population and is broadly representative of the Swedish population in terms of basic characteristics, suggesting that the results may be generalizable to Sweden as a whole. Although the inclusion of age, sex, education and socioeconomic group affected the main effect coefficients and the explanatory power modestly, the analyses revealed some interesting findings. TTO values were significantly positive for nearly all age groups and had a negative sign for the oldest indicating that age might be a further health indicator in addition to the five dimensions. TTO values were higher for women, while the coefficients for education did not reveal any significant differences. The coefficients for socioeconomic groups were positive and significant. Heterogeneity across surveys was observed. In the 2004 survey, anxiety/depression had greater impact and the N3 coefficient was greater; in the 2006 survey, usual activities had greater impact, on both TTO and VAS values. This may reflect the socio-demographic composition of the samples. Further research should investigate differences in valuation due to socio-demographic or other possible unobserved variables. The relatively high non-response rate for VAS in the 2004 survey is a matter of concern to which we have no explanation. However, the non-responders to VAS were similar to other non-responders.

The VAS scale was anchored between worst and best imaginable health which did not allow for anchoring between 0 (dead) and 1 (full health). Hence, these raw and estimated VAS values do not correspond to the 0–1 scale requirement for QALY calculations. We did not rescale the VAS values due to the ambiguity of where on the scale dead should be placed [56, 57]. If the VAS values should be used for QALY calculations, rescaling is necessary [24].

The observed and the predicted mean TTO value for the health state 11111 is 0.97. It is logical that this value is somewhat below 1, as individuals may have health problems in dimensions not covered by the EQ-5D. They may also have some health problems in the five dimensions that are not sufficiently severe to tick the ‘moderate problems box’ (with only three categories in a dimension, individuals will have to pick the category perceived as closest to their health state). It is in principle possible to rescale the predicted TTO values so that the health state 11111 is defined as 1. However, we do not recommend such rescaling as it would convert responses to a scale that differs from directly measured TTO values (and the two would thus not be directly comparable).

Our study also showed that TTO and VAS valuations were consistently related to SRH. In studies employing SRH as a measure of health status, our results can be used to apply cardinality on the ordinal responses to the SRH question [58–60].

Several authors have recently advocated the normative standpoint of using experience-based values [15, 34–36, 61, 62]. The experience-based values in our study are from population surveys, which is in line with recent thinking on valuing health states [12, 15] and could reduce some of the focusing effects that are likely to appear in hypothetical valuation studies [14, 15]. In a general population health survey, respondents may be more focused on their overall perceptions of their health status (and thus the valuation of the EQ-5D health state) without framing this perception into a particular disease condition or the actual dimensions and levels of the EQ-5D descriptive system. Our study also takes into consideration one argument for using so called social values; namely that health policies and intervention affect us all (as tax payers and potential patients), and therefore, values should be representative of the Swedish population, i.e., not representing a narrowly defined group of patients.

The normative question on whose values to use, or what value set, may have implications for economic evaluation and ultimately resource allocation [12, 39]. In our study, anxiety/depression has the greatest impact on both TTO and VAS values, as suggested in other studies [12, 14] followed by usual activities. The results underline the importance of mental health for individuals’ overall HRQoL. For TTO values, mobility has greater impact than pain/discomfort, whereas the opposite was seen for VAS values. If values are based on preferences for hypothetical health states, an intervention may seemingly lead to a greater gain than if values based on self-perceived health states are used, due to the lower values which are usually assigned when considering hypothetical health states. Whether this is an overestimation of the gain depends on whose preferences are considered most appropriate. The relative influence of HRQoL on QALY calculations is also affected by whose values are used.

Visual analogue scale (VAS) is not a choice-based method and the values were not anchored between dead and full health. Furthermore, there was an additional inconsistency (mobility) in the VAS model implying that more health states are not distinguished with the VAS value set. We therefore prefer the TTO value set. However, presentation of two value sets enables users to make their own judgement regarding which value set to use.

Swedish authors recommended the use of experience-based values in 1996 [18], and the Swedish reimbursement authority [41] recommended the use of experience-based values in 2003. Despite this fact, the UK TTO value set is predominantly used in Sweden. This may partly be due to lack of alternative value sets, and therefore, the results reported in this work represents a step towards value sets for EQ-5D health states that are based on Swedish experience-based values. The practical and normative implications of implementing the Swedish value sets in studies and subsequent health care decisions may warrant further discussion and investigation.

Furthermore, testing the performance of the value sets by assessing how the predicted values correspond to directly measured values in other populations is an interesting area for further research.

## Electronic supplementary material

Below is the link to the electronic supplementary material.

Supplementary material (PDF 1030 kb)
