# Supplementary material for: Swedish experience-based value sets for EQ-5D health states
Source: Qual Life Res. 2013 Aug 22;23(2):431–42. doi: 10.1007/s11136-013-0496-4 (PMC3967073; doi:10.1007/s11136-013-0496-4)
Supplement: Supplementary file 1 — Supplementary material (PDF 1030 kb) [file 11136_2013_496_MOESM1_ESM.pdf]

Online Resource (11136_2013_496_MOESM1_ESM.pdf)

JOURNAL:

Quality of Life Research (2013) DOI 10.1007/s11136-013-0496-4

TITLE:

SWEDISH EXPERIENCE-BASED VALUE SETS FOR EQ-5D HEALTH STATES

AUTHORS:

Kristina Burström [1, 2, 3]

Sun Sun [1, 3]

Ulf-G Gerdtham [4, 5, 6]

Martin Henriksson [7]

Magnus Johannesson [8]

Lars-Åke Levin [9]

Niklas Zethraeus [1, 10]

[1] Department of Learning, Informatics, Management and Ethics, Medical Management Centre, Karolinska Institutet, Tomtebodavägen 18A, 171 77 Stockholm, Sweden

[2] Department of Public Health Sciences, Equity and Health Policy Research Group, Karolinska Institutet, Tomtebodavägen 18A, 171 77 Stockholm, Sweden

[3] Stockholm County Council, Health Care Services, Tomtebodavägen 18A, 171 77 Stockholm, Sweden

[4] Department of Economics, Lund University, P.O. Box 7082, 220 07 Lund, Sweden

[5] Health Economics and Management, Institute of Economic Research, Lund University, P.O. Box 7082, 220 07 Lund, Sweden

[6] Centre for Primary Health Care Research, Lund University, P.O. Box 7082, 220 07 Lund, Sweden

[7] Department of Health Economics, AstraZeneca Nordic, 151 85 Södertälje, Sweden

[8] Department of Economics, Stockholm School of Economics, P.O Box 6501, 113 83 Stockholm, Sweden

[9] Department of Medical and Health Sciences, Center for Medical Technology Assessment, Linköping University, 581 83 Linköping, Sweden

[10] The Dental and Pharmaceutical Benefits Agency, P.O. Box 225 20, 104 22 Stockholm, Sweden

CORRESPONDING AUTHOR:
Kristina Burström
Associate Professor of Health Economics
Karolinska Institutet
Department of Learning, Informatics, Management and Ethics
Tomtebodavägen 18A, SE-171 77 Stockholm, Sweden
Email: kristina.burstrom@ki.se

TABLES AND FIGURE IN ONLINE RESOURCE

Supplementary Table S1 Socio-demographic characteristics of the populations 2004 and 2006, census data and data from the ULF survey

Supplementary Table S2 Characteristics of the respondents, by survey

Supplementary Table S3 Estimated TTO and VAS values for EQ-5D health states

Supplementary Table S4 Regression analysis on VAS values, EQ-5D dimensions

Supplementary Table S5 Correlation and Mean Absolute Difference (MAD), VAS values, EQ-5D

Supplementary Table S6 Regression analysis, comparison of TTO and VAS values, EQ-5D dimensions, main effect models, pooled data

Supplementary Table S7 Regression analysis on TTO and VAS values, EQ-5D dimensions, main effect models, comparison by survey

Supplementary Table S8 Regression analysis on TTO and VAS values, SRH

Supplementary Table S9 Estimated TTO and VAS values for SRH

Supplementary Table S10 Regression analysis, comparison of TTO and VAS values, SRH, main effect model, pooled data

Supplementary Table S11 Regression analysis on TTO and VAS values, SRH, main effect model, comparison by survey

Supplementary Fig. S1 Estimated VAS values predicted by different OLS models compared to the observed mean VAS values for health states with five or more observations (n=80)

DATA

Response rate, internal non-responders, exclusion criteria

Data consisted of responses from 51,254 individuals, aged 18–80 years. The response rates were similar in the surveys: 59% (in the 2004 survey) and 61% (in the 2006 survey) yielding a sample size of 27,963 individuals aged 18–80 years (2004 survey), and 23,291 individuals aged 18–64 years (2006 survey). Women had a higher response rate than men. After exclusion of 2,085 individuals (4%), who had missing answer on any of the EQ-5D dimensions, 49,169 individuals were included. The proportions of missing answers were 5% (2004 survey) and 3% (2006 survey).

Subsequently, 3,692 individuals (7.5%) were excluded from the TTO analyses due to missing TTO answer. The proportions of missing answers were 9.5% (2004 survey) and 5.1% (2006 survey). Thus, the pooled data set for the present TTO analyses consisted of 45,477 individuals. Similarly, 7,408 (15.1%) individuals were excluded from the VAS analyses due to missing answer on the VAS. The proportions of missing answers were 24.0% (2004 survey) and 4.5% (2006 survey). Thus, the pooled data set for the present VAS analyses consisted of 41,761 individuals. Before carrying out analyses on the SRH question individuals with missing answer on SRH were excluded: 741 individuals for the TTO analyses and 637 individuals for the VAS analyses.

The internal non-responders to the TTO question (7.5%) had a significantly higher mean age and worse health status compared to those who had answer the TTO question. Non-response to the TTO question was more common among women than among men and in groups with lower education and among manual workers. The same pattern was seen in non-responders to the VAS question (15.1%) except that there was no difference in non-response rate between men and women. Due to the higher proportion of non-responders to the VAS question in the 2004 survey we examined the two surveys separately with respect to VAS, resulting in the same pattern as in the pooled data.

However, in the 2004 survey the non-response rate was higher among women than men, as found in the analyses of non-responders to the TTO question for the pooled data.

Categorisation of age, educational level and socioeconomic group

Age was categorised in the age groups: 18–24; 25–34; 35–44; 45–54; 55–64; 65–74; 75–88 years (reference group 18–24 years). Educational level was categorized into low (elementary school 9–10 years); medium (secondary school 3–4 years); high (more than 3–4 years secondary school). Low education was used as reference group. Respondents were categorized into six socioeconomic groups: unskilled manual; skilled manual; lower non-manual; intermediate non-manual; higher non-manual; self-employed and farmers (reference group unskilled manual workers).

Test for parameter homogeneity across surveys

The estimated models were tested for homogeneity in the parameters of the regressors across the two surveys of the pooled model, with the age restricted to 18–64 years, i.e. the intercept and the slope of all regressors. In this F-test analysis, we hypothesised that the intercepts were equal in the two surveys. Given rejection of this hypothesis we secondly tested the hypothesis that the parameters of all socio-demographic regressors were equal. Given rejection of this hypothesis we finally tested the hypothesis that the parameters of all the dimensional regressors (mobility; self-care; usual activities; pain/discomfort; anxiety/depression) were equal.

SUPPLEMENTARY RESULTS

Regression analysis on VAS values for EQ-5D dimensions

Results of the regression analysis on individual VAS values for EQ-5D dimensions are presented in Supplementary Table S4.

The VAS results were consistent with lower values the more severe the health state was (Model 1), except for self-care and mobility where the value for level 3 was not lower compared to level 2. For the self-care dimension the coefficient for level 3 was positive, but not significant. The N3 variable had a negative sign and was significant (Model 2). The results when entering the MO23 and SC23 variables (merged levels 2 and 3 for mobility and self-care) are shown in Model 3 and Model 4 (including N3).

For health states with five or more observations the Spearman correlation coefficient was greatest for Model 3 and MAD was smallest for Model 4 (Supplementary Table S5). For health states with ten or more observations the correlation was highest and MAD was smallest in Model 4. The adjusted R^2 was similar for all models (around 0.48) (Supplementary Table S4).

Supplementary Figure S1 shows the estimated VAS values predicted by the different OLS models compared to the observed mean VAS values for health states with five or more observations.

Looking at the main effects (Model 4), severe problems in the anxiety/depression dimension had the greatest coefficient (23.7), followed by usual activities (15.0) and pain/discomfort (12.9) (Supplementary Table S4). For moderate problems, the greatest coefficient was for usual activities (12.1) followed by anxiety/depression (10.0) and pain/discomfort (6.7). The merged coefficients for mobility (9.8) and self-care (0.8) represented any move away from no problems. The difference

between the predicted and the observed mean values exceeded 10 for 14% of the health states with five or more observations.

The effect of age, sex, education and socioeconomic group on VAS values followed the same tendency as the effect on TTO values.

A Swedish VAS value set, based on Model 4, for the 243 health states, is presented in Supplementary Table S3.

Comparison of TTO and VAS values for Models 2 and 4 for EQ-5D is presented in Supplementary Table S6 (pooled data) and Table S7 (by survey).

Regression analysis on TTO values and VAS values for SRH

Results of the regression analysis on individual TTO and VAS values for SRH levels are presented in Supplementary Table S8. Both TTO and VAS were consistent and significant with lower values the poorer the level of SRH. The effect of socio-demographic variables was similar to the results for EQ-5D dimensions, except the coefficient for sex on the VAS values that was negative.

The TTO and VAS values, estimated based on Model 1, for the different severity levels for SRH, are presented in Supplementary Table S9. These results can be used to convert SRH to TTO and VAS values in studies that include information on SRH.

Comparison of TTO and VAS values for SRH levels is presented in Supplementary Table S10 (pooled data) and Table S11 (by survey).

Split sample test

The results for the Spearman correlation coefficients and the MAD in the split sample test were similar to those obtained in the whole sample. For the TTO model, for health states with five or more observations, the Spearman correlation coefficient remained 0.83 and the MAD was still below 0.06. For the VAS model, for health states with five or more observations, the Spearman correlation coefficient was 0.93, and the MAD was 5.0, similar to that of the whole sample. For health states with ten or more observations, the results from the split sample test showed greater Spearman correlation coefficients and smaller MAD, compared to health states with five or more observations, which was similar to the findings from the whole sample.

Homogeneity across surveys

The results of the F-tests showed that homogeneity of the regression coefficients across surveys were rejected irrespectively of whether TTO or VAS were considered or inclusion of socio-demographic control factors; indicating that the effects of the regressors, both socio-demographic and dimensional regressors, on TTO and VAS values were not equal across the two surveys (except for dimensional regressors on TTO values in the SRH regression) (Table 3 main manuscript; Supplementary Tables S4 and S8). It is likely that this heterogeneity is mainly due to regional heterogeneity since there is only a two-year time difference between the surveys in the different areas. In the TTO regression, the N3 variable was smaller and non-significant in the 2006 survey and the greatest difference was seen in the anxiety/depression dimension (Supplementary Table S7). In the VAS regression, the differences were similar.

Supplementary Table S1 Socio-demographic characteristics of the populations 2004 and 2006, census data and data from ULF survey

	2004		2006	
	Scania Region	Sweden	Stockholm County	Sweden
**Proportion of whole population (%) [a]**	12.9	n.a.	21.0	n.a.
**Living outside localities (%) [a]**	[e]12.3	[e]15.6	[e]4.6	[e]15.6
**Women (%) [a]**	50.8	50.4	50.8	50.4
**Mean age (years) [a]**	40.9	40.8	39.1	40.9
**Married (%) 20-64 years [a]**	45.6	43.5	40.7	43.0
**Educational level (%) 20-64 years [a]**				
Elementary school (9-10 years)	17.8	17.1	13.8	15.9
Secondary school 3-4 years	46.7	48.6	41.8	48.2
More than 3-4 years secondary school	33.7	32.9	42.0	34.1
**Mean income (SEK [b]) 20-64 years [a]**	212,500	227,900	275,400	242,000
**Socioeconomic group (%) 20-64 years [c]**				
Unskilled manual workers	16.3	17.7	11.0	17.4
Skilled manual workers	14.1	12.7	8.8	14.4
Lower non-manual workers	10.5	10.9	11.8	11.4
Intermediate non-manual workers	17.3	18.6	21.7	18.7
Higher non-manual workers	13.7	13.1	22.2	13.0
Self-employed and farmers	7.0	7.7	8.8	7.5
**Less than good SRH (%) 20-64 years [c,d]**	19.5	23.1	19.3	21.7
**Proportion of whole population 20-64 years [c]**	12.3	n.a.	20.6	n.a.
**Women (%) 20-64 years [c]**	50.1	49.7	52.4	50.5
**Mean age (years) 20-64 years [c]**	42.0	42.7	42.6	43.3

[a] Census data
[b] Current prices (Swedish Kronor) SEK 1 = Euro 0.109 (2004, 2006)
[c] Data from Statistics Sweden's Survey of Living Conditions (ULF)
[d] *SRH* self-rated health
[e] Data from 2005

Supplementary Table S2 Characteristics of the respondents, by survey

	**2004 survey**		**2006 survey**	
	**18-80 years (n=26,627)**		**18-64 years (n=22,542)**	
**Variable**	**%**	**n**	**%**	**n**
**Women**	54.7	14,553	58.3	13,147
**Mean age**	48.5 years	26,627	43.4 years	22,542
**Age group**				
18-24 years	9.2	2,443	9.0	2,040
25-34 years	15.1	4,017	18.7	4,222
35-44 years	17.9	4,779	24.5	5,516
45-54 years	18.1	4,811	22.2	4,993
55-64 years	20.0	5,337	25.6	5,771
65-74 years	13.9	3,692	n.a.	n.a.
75-80 years	5.8	1,548	n.a.	n.a.
**Educational level**				
Low	20.3	5,420	13.3	2,994
Medium	42.1	11,200	42.2	9,503
High	31.9	8,489	42.9	9,683
Missing	5.7	1,518	1.6	362
**Socioeconomic group**				
Unskilled manual	19.6	5,227	15.8	3,561
Skilled manual	12.2	3,238	12.0	2,711
Lower non-manual	8.7	2,312	13.5	3,050
Intermediate non-manual	15.0	3,989	23.1	5,197
Higher non-manual	9.6	2,569	18.6	4,182
Self-employed and farmers	1.7	450	6.8	1,539
Other	33.2	8,842	10.21	2,302
**Less than good SRH**	30.0	7,994	24.8	5,599
**Mobility**				
Moderate problems (level 2)	11.0	2,924	8.5	1,916
Severe problems (level 3)	0.1	24	0.1	26
**Self-care**				
Moderate problems (level 2)	1.1	294	1.4	306
Severe problems (level 3)	0.5	129	0.3	69
**Usual activities**				
Moderate problems (level 2)	6.7	1,785	8.9	2,000
Severe problems (level 3)	1.0	276	1.2	260
**Pain/discomfort**				
Moderate problems (level 2)	50.1	13,351	39.2	8,834
Severe problems (level 3)	4.7	1,259	3.5	779
**Anxiety/depression**				
Moderate problems (level 2)	30.4	8,080	31.3	7,046
Severe problems (level 3)	2.5	677	2.9	645
**Problems in at least one EQ-5D dimension**	63.8	16,998	56.0	12,620
**Problems on level 3**	7.2	1,916	6.1	1,371
**TTO (mean)**	0.90	24,090	0.92	21,387
**EQ VAS (mean)**	79.1	20,229	79.9	21,532

TTO time trade off, *VAS* visual analogue scale, *SRH* self-rated health

Supplementary Table S3 Estimated TTO and VAS values for EQ-5D health states

Obs	EQ-5D	TTO value	VAS value	Obs	EQ-5D	TTO value	VAS value	Obs	EQ-5D	TTO value	VAS value
1	11111	0.9694	88.86	82	21111	0.9028	79.10	163	31111	0.8015	69.65
2	11112	0.9142	78.90	83	21112	0.8477	69.13	164	31112	0.7463	59.69
3	11113	0.7185	55.69	84	21113	0.6519	45.93	165	31113	0.5938	45.93
4	11121	0.9349	82.16	85	21121	0.8683	72.39	166	31121	0.7669	62.94
5	11122	0.8797	72.19	86	21122	0.8131	62.43	167	31122	0.7117	52.98
6	11123	0.6839	48.99	87	21123	0.6174	39.22	168	31123	0.5593	39.22
7	11131	0.8357	66.51	88	21131	0.7691	56.75	169	31131	0.7110	56.75
8	11132	0.7805	56.55	89	21132	0.7139	46.78	170	31132	0.6558	46.78
9	11133	0.6280	42.79	90	21133	0.5615	33.02	171	31133	0.5034	33.02
10	11211	0.8682	76.75	91	21211	0.8016	66.99	172	31211	0.7002	57.54
11	11212	0.8130	66.79	92	21212	0.7464	57.02	173	31212	0.6450	47.58
12	11213	0.6172	43.58	93	21213	0.5507	33.82	174	31213	0.4926	33.82
13	11221	0.8336	70.04	94	21221	0.7671	60.28	175	31221	0.6657	50.83
14	11222	0.7784	60.08	95	21222	0.7119	50.32	176	31222	0.6105	40.87
15	11223	0.5827	36.88	96	21223	0.5161	27.11	177	31223	0.4580	27.11
16	11231	0.7345	54.40	97	21231	0.6679	44.64	178	31231	0.6098	44.64
17	11232	0.6793	44.44	98	21232	0.6127	34.67	179	31232	0.5546	34.67
18	11233	0.5268	30.68	99	21233	0.4602	20.91	180	31233	0.4021	20.91
19	11311	0.7906	64.42	100	21311	0.7241	54.65	181	31311	0.6660	54.65
20	11312	0.7354	54.45	101	21312	0.6689	44.69	182	31312	0.6108	44.69
21	11313	0.5830	40.69	102	21313	0.5164	30.93	183	31313	0.4583	30.93
22	11321	0.7561	57.71	103	21321	0.6895	47.94	184	31321	0.6314	47.94
23	11322	0.7009	47.75	104	21322	0.6343	37.98	185	31322	0.5762	37.98
24	11323	0.5484	33.99	105	21323	0.4819	24.22	186	31323	0.4238	24.22
25	11331	0.7002	51.51	106	21331	0.6336	41.75	187	31331	0.5755	41.75
26	11332	0.6450	41.55	107	21332	0.5785	31.78	188	31332	0.5204	31.78
27	11333	0.4925	27.79	108	21333	0.4260	18.02	189	31333	0.3679	18.02
28	12111	0.9418	88.08	109	22111	0.8752	78.31	190	32111	0.7738	68.86
29	12112	0.8866	78.11	110	22112	0.8200	68.35	191	32112	0.7187	58.90
30	12113	0.6908	54.91	111	22113	0.6243	45.14	192	32113	0.5662	45.14
31	12121	0.9072	81.37	112	22121	0.8407	71.60	193	32121	0.7393	62.16
32	12122	0.8521	71.41	113	22122	0.7855	61.64	194	32122	0.6841	52.19
33	12123	0.6563	48.20	114	22123	0.5897	38.43	195	32123	0.5316	38.43
34	12131	0.8081	65.73	115	22131	0.7415	55.96	196	32131	0.6834	55.96
35	12132	0.7529	55.76	116	22132	0.6863	46.00	197	32132	0.6282	46.00
36	12133	0.6004	42.00	117	22133	0.5339	32.24	198	32133	0.4758	32.24
37	12211	0.8406	75.97	118	22211	0.7740	66.20	199	32211	0.6726	56.75
38	12212	0.7854	66.00	119	22212	0.7188	56.24	200	32212	0.6174	46.79
39	12213	0.5896	42.80	120	22213	0.5231	33.03	201	32213	0.4650	33.03
40	12221	0.8060	69.26	121	22221	0.7395	59.49	202	32221	0.6381	50.05
41	12222	0.7508	59.30	122	22222	0.6843	49.53	203	32222	0.5829	40.08
42	12223	0.5551	36.09	123	22223	0.4885	26.32	204	32223	0.4304	26.32
43	12231	0.7068	53.62	124	22231	0.6403	43.85	205	32231	0.5822	43.85
44	12232	0.6517	43.65	125	22232	0.5851	33.89	206	32232	0.5270	33.89
45	12233	0.4992	29.89	126	22233	0.4326	20.13	207	32233	0.3745	20.13
46	12311	0.7630	63.63	127	22311	0.6965	53.86	208	32311	0.6384	53.86
47	12312	0.7078	53.67	128	22312	0.6413	43.90	209	32312	0.5832	43.90
48	12313	0.5554	39.91	129	22313	0.4888	30.14	210	32313	0.4307	30.14
49	12321	0.7285	56.92	130	22321	0.6619	47.16	211	32321	0.6038	47.16
50	12322	0.6733	46.96	131	22322	0.6067	37.19	212	32322	0.5486	37.19
51	12323	0.5208	33.20	132	22323	0.4543	23.43	213	32323	0.3962	23.43
52	12331	0.6726	50.73	133	22331	0.6060	40.96	214	32331	0.5479	40.96
53	12332	0.6174	40.76	134	22332	0.5508	31.00	215	32332	0.4927	31.00
54	12333	0.4649	27.00	135	22333	0.3984	17.24	216	32333	0.3403	17.24
55	13111	0.8985	78.63	136	23111	0.8319	68.86	217	33111	0.7738	68.86
56	13112	0.8433	68.67	137	23112	0.7768	58.90	218	33112	0.7187	58.90
57	13113	0.6908	54.91	138	23113	0.6243	45.14	219	33113	0.5662	45.14
58	13121	0.8640	71.92	139	23121	0.7974	62.16	220	33121	0.7393	62.16
59	13122	0.8088	61.96	140	23122	0.7422	52.19	221	33122	0.6841	52.19
60	13123	0.6563	48.20	141	23123	0.5897	38.43	222	33123	0.5316	38.43
61	13131	0.8081	65.73	142	23131	0.7415	55.96	223	33131	0.6834	55.96
62	13132	0.7529	55.76	143	23132	0.6863	46.00	224	33132	0.6282	46.00
63	13133	0.6004	42.00	144	23133	0.5339	32.24	225	33133	0.4758	32.24
64	13211	0.7973	66.52	145	23211	0.7307	56.75	226	33211	0.6726	56.75
65	13212	0.7421	56.56	146	23212	0.6755	46.79	227	33212	0.6174	46.79
66	13213	0.5896	42.80	147	23213	0.5231	33.03	228	33213	0.4650	33.03
67	13221	0.7627	59.81	148	23221	0.6962	50.05	229	33221	0.6381	50.05
68	13222	0.7075	49.85	149	23222	0.6410	40.08	230	33222	0.5829	40.08
69	13223	0.5551	36.09	150	23223	0.4885	26.32	231	33223	0.4304	26.32
70	13231	0.7068	53.62	151	23231	0.6403	43.85	232	33231	0.5822	43.85
71	13232	0.6517	43.65	152	23232	0.5851	33.89	233	33232	0.5270	33.89
72	13233	0.4992	29.89	153	23233	0.4326	20.13	234	33233	0.3745	20.13
73	13311	0.7630	63.63	154	23311	0.6965	53.86	235	33311	0.6384	53.86
74	13312	0.7078	53.67	155	23312	0.6413	43.90	236	33312	0.5832	43.90
75	13313	0.5554	39.91	156	23313	0.4888	30.14	237	33313	0.4307	30.14
76	13321	0.7285	56.92	157	23321	0.6619	47.16	238	33321	0.6038	47.16
77	13322	0.6733	46.96	158	23322	0.6067	37.19	239	33322	0.5486	37.19
78	13323	0.5208	33.20	159	23323	0.4543	23.43	240	33323	0.3962	23.43
79	13331	0.6726	50.73	160	23331	0.6060	40.96	241	33331	0.5479	40.96
80	13332	0.6174	40.76	161	23332	0.5508	31.00	242	33332	0.4927	31.00
81	13333	0.4649	27.00	162	23333	0.3984	17.24	243	33333	0.3403	17.24

Supplementary Table S4 Regression analysis on VAS values, EQ-5D dimensions *(two pages)*

Variable	Model 1 Estimate	p-value	Model 2 Estimate	p-value	Model 3 Estimate	p-value	Model 4 Estimate	p-value	Model 5 Estimate	p-value	Model 6 Estimate	p-value	Model 7 Estimate	p-value
**Intercept**	88.83	<0.0001	88.85	<0.0001	88.84	<0.0001	88.86	<0.0001	88.87	<0.0001	88.44	<0.0001	88.00	<0.0001
**Mobility**														
Level 2	-9.88	<0.0001	-9.75	<0.0001	-	-	-	-	-	-	-	-	-	-
Level 3	-2.72	0.5911	-2.45	0.6157	-	-	-	-	-	-	-	-	-	-
Level 2 and 3	-	-	-	-	-9.86	<0.0001	-9.77	<0.0001	-9.65	<0.0001	-9.64	<0.0001	-9.56	<0.0001
**Self-care**														
Level 2	-3.75	0.0001	-3.53	0.0002	-	-	-	-	-	-	-	-	-	-
Level 3	0.58	0.7332	7.82	<0.0001	-	-	-	-	-	-	-	-	-	-
Level 2 and 3	-	-	-	-	-2.38	0.0060	-0.79	0.3846	-0.68	0.4514	-0.63	0.4879	-0.57	0.5252
**Usual activities**														
Level 2	-12.28	<0.0001	-11.85	<0.0001	-12.37	<0.0001	-12.11	<0.0001	-12.21	<0.0001	-12.24	<0.0001	-12.20	<0.0001
Level 3	-18.01	<0.0001	-15.33	<0.0001	-17.45	<0.0001	-15.00	<0.0001	-15.01	<0.0001	-15.03	<0.0001	-15.03	<0.0001
**Pain/discomfort**														
Level 2	-6.73	<0.0001	-6.71	<0.0001	-6.73	<0.0001	-6.71	<0.0001	-6.72	<0.0001	-6.76	<0.0001	-6.69	<0.0001
Level 3	-20.00	<0.0001	-10.94	<0.0001	-20.11	<0.0001	-12.90	<0.0001	-12.94	<0.0001	-12.99	<0.0001	-12.86	<0.0001
**Anxiety/depression**														
Level 2	-10.04	<0.0001	-9.93	<0.0001	-10.05	<0.0001	-9.96	<0.0001	-9.97	<0.0001	-10.05	<0.0001	-10.03	<0.0001
Level 3	-30.52	<0.0001	-22.07	<0.0001	-30.52	<0.0001	-23.72	<0.0001	-23.75	<0.0001	-23.80	<0.0001	-23.72	<0.0001
**N3**	-	-	-11.73	<0.0001	-	-	-9.45	<0.0001	-9.47	<0.0001	-9.49	<0.0001	-9.47	<0.0001
**Age group** [a]														
25-34	-	-	-	-	-	-	-	-	0.01	0.9766	0.00	0.9968	-0.42	0.1220
35-44	-	-	-	-	-	-	-	-	-0.44	0.0867	-0.42	0.1066	-0.91	0.0008
45-54	-	-	-	-	-	-	-	-	0.43	0.0974	0.47	0.0682	0.00	0.9950
55-64	-	-	-	-	-	-	-	-	0.34	0.1966	0.40	0.1222	-0.06	0.8355
65-74	-	-	-	-	-	-	-	-	0.02	0.9434	0.10	0.7660	-0.14	0.6747
75-80	-	-	-	-	-	-	-	-	-3.09	<0.0001	-3.00	<0.0001	-2.90	<0.0001
**Sex** [b]	-	-	-	-	-	-	-	-	-	-	0.79	<0.0001	0.74	<0.0001

**Educational level** [c]														
Medium	-	-	-	-	-	-	-	-	-	-	-	-	0.35	0.1063
High	-	-	-	-	-	-	-	-	-	-	-	-	0.34	0.1454
Missing	-	-	-	-	-	-	-	-	-	-	-	-	-0.19	0.7604
**Socioeconomic group** [d]														
Skilled manual	-	-	-	-	-	-	-	-	-	-	-	-	0.56	0.0292
Lower non-manual	-	-	-	-	-	-	-	-	-	-	-	-	0.88	0.0006
Intermed non-manual	-	-	-	-	-	-	-	-	-	-	-	-	0.99	<0.0001
Higher non-manual	-	-	-	-	-	-	-	-	-	-	-	-	0.72	0.0037
Self-employed	-	-	-	-	-	-	-	-	-	-	-	-	1.32	0.0001
Other	-	-	-	-	-	-	-	-	-	-	-	-	0.04	0.8750
**Observations**	41,761		41,761		41,761		41,761		41,761		41,761		41,761	
**Adjusted R^2**	0.4845		0.4875		0.4841		0.4864		0.4873		0.4877		0.4882	
**F statistics** [e, f]	$F_{(1,38111)}$=32.78 *		$F_{(1,38110)}$=31.05 *		$F_{(1,38113)}$=32.15 *		$F_{(1,38112)}$=30.02 *		$F_{(1,38112)}$=30.02 *		$F_{(1,38112)}$=30.02 *		$F_{(1,38112)}$=30.02 *	
**F statistics** [e, g]									$F_{(88,38104)}$=3.56 *		$F_{(20,38102)}$=5.62 *		$F_{(28,38084)}$=3.54 *	
**F statistics** [e, h]	$F_{(21,38101)}$=42.35 *		$F_{(22,38100)}$=25.59 *		$F_{(8,38105)}$=49.93 *		$F_{(9,38103)}$=46.24 *		$F_{(27,38095)}$=45.86 *		$F_{(29,38093)}$=46.12 *		$F_{(9,38075)}$=47.63 *	

[a] Reference group: 18-24 years
[b] Reference group: men
[c] Reference group: low educational level
[d] Reference group: unskilled manual workers
[e] Age restricted to 18-64 years
[f] F-test of equal intercepts in the two surveys
[g] F-test of equal parameters of all non-dimensional regressors in the two surveys
[h] F-test of equal parameters of all dimensional regressors in the two surveys
[*] p=0.001
VAS visual analogue scale

Supplementary Table S5 Correlation and Mean Absolute Difference (MAD), VAS values, EQ-5D

Number of health states	VAS							
	**Model 1**		**Model 2**		**Model 3**		**Model 4**	
	**Correlation**	**MAD**	**Correlation**	**MAD**	**Correlation**	**MAD**	**Correlation**	**MAD**
**n>=5**	0.942	5.11	0.942	4.49	0.929	5.40	0.919	5.22
**n>=10**	0.957	4.42	0.967	3.57	0.955	4.49	0.960	3.81

VAS visual analogue scale

Supplementary Table S6 Regression analysis, comparison of TTO and VAS values, EQ-5D dimensions, main effect models, pooled data

Variable	TTO				VAS			
	**Model 2**		**Model 4**		**Model 2**		**Model 4**	
	**Estimate**	**p-value**	**Estimate**	**p-value**	**Estimate**	**p-value**	**Estimate**	**p-value**
**Intercept**	0.9716	<0.0001	0.9717	<0.0001	88.94	<0.0001	88.95	<0.0001
**Mobility**								
Level 2	-0.0652	<0.0001	-0.0663	<0.0001	-9.53	<0.0001	-	-
Level 3	-0.1448	0.0026	-0.1060	0.0216	-3.58	0.5155	-	-
Level 2 and 3							-9.57	<0.0001
**Self-care**								
Level 2	-0.0588	<0.0001	-	-	-3.51	0.0008	-	-
Level 3	0.0772	0.0014	-	-	8.29	<0.0001	-	-
Level 2 and 3	-	-	-0.0291	0.0242	-	-	-0.54	0.5851
**Usual activities**								
Level 2	-0.1012	<0.0001	-0.1038	<0.0001	-12.06	<0.0001	-12.32	<0.0001
Level 3	-0.1331	<0.0001	-0.1356	<0.0001	-15.21	<0.0001	-14.99	<0.0001
**Pain/discomfort**								
Level 2	-0.0336	<0.0001	-0.0336	<0.0001	-6.53	<0.0001	-6.53	<0.0001
Level 3	-0.0676	<0.0001	-0.0888	<0.0001	-10.65	<0.0001	-12.59	<0.0001
**Anxiety/depression**								
Level 2	-0.0532	<0.0001	-0.0535	<0.0001	-9.88	<0.0001	-9.91	<0.0001
Level 3	-0.1904	<0.0001	-0.2094	<0.0001	-22.15	<0.0001	-23.83	<0.0001
**N3**	-0.0779	<0.0001	-0.0525	0.0005	-12.08	<0.0001	-9.78	<0.0001
**Observations**	39,527		39,527		39,527		39,527	
**Adjusted R^2**	0.2536		0.2523		0.4783		0.4772	

TTO time tradeoff, *VAS* visual analogue scale

Supplementary Table S7 Regression analysis on TTO and VAS values, EQ-5D dimensions, main effect models, comparison by survey

	TTO								VAS									
	Model 2				Model 4				Model 2				Model 4					
	2004 Survey		2006 Survey		2004 Survey		2006 Survey		2004 Survey		2006 Survey		2004 Survey [a]				2006 Survey	
Variable	Estimate	p-value	Estimate	p-value	Estimate	p-value	Estimate	p-value	Estimate	p-value	Estimate	p-value	Estimate	p-value	Estimate	p-value	Estimate	p-value
Intercept	0.9639	<0.0001	0.9741	<0.0001	0.9640	<0.0001	0.9741	<0.0001	89.05	<0.0001	88.63	<0.0001	89.07	<0.0001	89.07	<0.0001	88.64	<0.0001
**Mobility**																		
Level 2	-0.0712	<0.0001	-0.0561	<0.0001	-0.0717	<0.0001	-0.0570	<0.0001	-10.80	<0.0001	-8.55	<0.0001	-	-	-	-	-	-
Level 3	-0.1532	0.0168	-0.1590	0.0068	-0.1225	0.0520	-0.1325	0.0200	2.47	0.7405	-6.99	0.2765	-	-	-	-	-	-
Level 2 and 3									-	-	-	-	-10.81	<0.0001	-10.80	<0.0001	-8.60	<0.0001
**Self-care**																		
Level 2	-0.0471	0.0083	-0.0543	0.0030	-	-	-	-	-3.90	0.0069	-4.30	0.0005	-	-	-	-	-	-
Level 3	0.0421	0.1795	0.0532	0.0889	-	-	-	-	9.01	0.0004	7.51	0.0079	-	-	-	-	-	-
Level 2 and 3	-	-	-	-	-0.0249	0.1182	-0.0349	0.0345	-	-	-	-	0.25	0.8597	-	-	-2.25	0.0549
**Usual activities**																		
Level 2	-0.0979	<0.0001	-0.1111	<0.0001	-0.0998	<0.0001	-0.1128	<0.0001	-12.24	<0.0001	-12.55	<0.0001	-12.60	<0.0001	-12.58	<0.0001	-12.74	<0.0001
Level 3	-0.1195	<0.0001	-0.1628	<0.0001	-0.1235	<0.0001	-0.1635	<0.0001	-15.40	<0.0001	-16.90	<0.0001	-14.30	<0.0001	-14.26	<0.0001	-17.00	<0.0001
**Pain/discomfort**																		
Level 2	-0.0339	<0.0001	-0.0303	<0.0001	-0.0339	<0.0001	-0.0302	<0.0001	-6.68	<0.0001	-6.58	<0.0001	-6.69	<0.0001	-6.69	<0.0001	-6.56	<0.0001
Level 3	-0.0725	0.0005	-0.0703	0.0005	-0.0895	<0.0001	-0.0830	<0.0001	-8.25	<0.0001	-12.00	<0.0001	-10.66	<0.0001	-10.70	<0.0001	-13.59	<0.0001
**Anxiety/depression**																		
Level 2	-0.0611	<0.0001	-0.0494	<0.0001	-0.0613	<0.0001	-0.0496	<0.0001	-11.05	<0.0001	-9.00	<0.0001	-11.09	<0.0001	-11.09	<0.0001	-9.02	<0.0001
Level 3	-0.2116	<0.0001	-0.1846	<0.0001	-0.2262	<0.0001	-0.1962	<0.0001	-26.74	<0.0001	-18.70	<0.0001	-28.62	<0.0001	-28.68	<0.0001	-20.11	<0.0001
**N3**	-0.0706	0.0013	-0.0403	0.0603	-0.0507	0.0075	-0.0247	0.2125	-15.14	<0.0001	-8.12	<0.0001	-12.46	<0.0001	-12.39	<0.0001	-6.19	<0.0001
**Observations**	24,090		21,387		24,090		21,387		20,229		21,532		20,229		20,229		21,532	
**Adjusted R^2**	0.2372		0.2441		0.2367		0.2433		0.5186		0.4642		0.5169		0.5169		0.4633	

[a] We present an alternative Model 4 for the 2004 survey without positive and non-significant the coefficient for self-care level 2 and 3

Supplementary Table S8 Regression analysis on TTO and VAS values, SRH *(two pages)*

	TTO								VAS							
	**Model 1 SRH**		**Model 2 SRH**		**Model 3 SRH**		**Model 4 SRH**		**Model 1 SRH**		**Model 2 SRH**		**Model 3 SRH**		**Model 4 SRH**	
**Variable**	**Estimate**	**p-value**	**Estimate**	**p-value**	**Estimate**	**p-value**	**Estimate**	**p-value**	**Estimate**	**p-value**	**Estimate**	**p-value**	**Estimate**	**p-value**	**Estimate**	**p-value**
**Intercept**	0.9751	<0.0001	0.9623	<0.0001	0.9589	<0.0001	0.9559	<0.0001	92.16	<0.0001	91.51	<0.0001	91.87	<0.0001	92.09	<0.0001
**SRH** [a]																
Good health	-0.0309	<0.0001	-0.0314	<0.0001	-0.0315	<0.0001	-0.0305	<0.0001	-8.80	<0.0001	-8.92	<0.0001	-8.91	<0.0001	-8.92	<0.0001
Fair health	-0.1402	<0.0001	-0.1411	<0.0001	-0.1414	<0.0001	-0.1389	<0.0001	-26.53	<0.0001	-26.84	<0.0001	-26.81	<0.0001	-26.81	<0.0001
Poor health	-0.3166	<0.0001	-0.3184	<0.0001	-0.3189	<0.0001	-0.3152	<0.0001	-50.52	<0.0001	-50.87	<0.0001	-50.81	<0.0001	-50.76	<0.0001
Very poor health	-0.4792	<0.0001	-0.4811	<0.0001	-0.4817	<0.0001	-0.4768	<0.0001	-64.11	<0.0001	-64.43	<0.0001	-64.38	<0.0001	-64.30	<0.0001
**Age group** [b]																
25-34	-	-	0.0102	0.0007	0.0101	0.0007	0.0042	0.1814	-	-	0.06	0.8244	0.06	0.8024	-0.17	0.5182
35-44	-	-	0.0107	0.0003	0.0109	0.0002	0.0039	0.2120	-	-	0.05	0.8386	0.03	0.9050	-0.28	0.2817
45-54	-	-	0.0176	<0.0001	0.0179	<0.0001	0.0111	0.0004	-	-	1.03	<0.0001	0.99	<0.0001	0.68	0.0089
55-64	-	-	0.0230	<0.0001	0.0235	<0.0001	0.0164	<0.0001	-	-	1.79	<0.0001	1.73	<0.0001	1.40	<0.0001
65-74	-	-	0.0188	<0.0001	0.0193	<0.0001	0.0149	<0.0001	-	-	2.40	<0.0001	2.34	<0.0001	2.11	<0.0001
75-80	-	-	-0.0255	<0.0001	-0.0248	<0.0001	-0.0146	0.0565	-	-	0.41	0.4268	0.33	0.5233	0.66	0.3113
**Sex** [c]	-	-	-	-	0.0058	0.0001	0.0053	0.0005	-	-	-	-	-0.62	<0.0001	-0.65	<0.0001

**Educational level** [d]																
Medium	-	-	-	-	-	-	0.0000	0.9992	-	-	-	-	-	-	-0.07	0.7331
High	-	-	-	-	-	-	0.0009	0.7468	-	-	-	-	-	-	-0.39	0.0820
Missing	-	-	-	-	-	-	-0.0171	0.0105	-	-	-	-	-	-	-0.73	0.2073
**Socioeconomic group** [e]																
Skilled manual	-	-	-	-	-	-	0.0080	0.0061	-	-	-	-	-	-	0.60	0.0184
Lower non-manual	-	-	-	-	-	-	0.0115	<0.0001	-	-	-	-	-	-	0.54	0.0303
Intermed non-manual	-	-	-	-	-	-	0.0137	<0.0001	-	-	-	-	-	-	0.75	0.0007
Higher non-manual	-	-	-	-	-	-	0.0168	<0.0001	-	-	-	-	-	-	0.35	0.1340
Self-employed	-	-	-	-	-	-	0.0137	0.0005	-	-	-	-	-	-	0.49	0.1226
Other	-	-	-	-	-	-	0.0000	0.9909	-	-	-	-	-	-	-0.44	0.0524
**Observations**	44,736		44,736		44,736		44,736		41,124		41,124		41,124		41,124	
**Adjusted R^2**	0.2261		0.2287		0.2290		0.2303		0.5304		0.5323		0.5326		0.5330	
**F statistics** [e, f]	$F_{(1,40603)}$=53.37 *		$F_{(1,40603)}$=53.37 *		$F_{(1,40603)}$=53.37 *		$F_{(1,40603)}$=53.37 *		$F_{(1,37611)}$=15.51 *		$F_{(1,37611)}$=15.51 *		$F_{(1,37611)}$=15.51 *		$F_{(1,37611)}$=15.51 *	
**F statistics** [e, g]			$F_{(13,40595)}$=12.74 *		$F_{(15,40595)}$=11.78 *		$F_{(28,40575)}$=6.44 *				$F_{(13,37603)}$=23.16 *		$F_{(15,37601)}$=21.40 *		$F_{(28,37583)}$=9.39 *	
**F statistics** [e, h]	$F_{(9,40599)}$=2.03 **		$F_{(17,40591)}$=1.79 **		$F_{(19,40589)}$=1.76 **		$F_{(4,40571)}$=1.56		$F_{(9,37609)}$=16.49 *		$F_{(17,37599)}$=17.44 *		$F_{(19,37597)}$=17.36 *		$F_{(4,37579)}$=18.73 *	

[a] Reference group: very good SRH
[b] Reference group: 18-24 years
[c] Reference group: men
[c] Reference group: low educational level
[e] Reference group: unskilled manual workers
[f] Age restricted to 18-64 years
[g] F-test of equal intercepts in the two surveys
[h] F-test of equal parameters of all non-dimensional regressors in the two surveys
[i] F-test of equal parameters of all dimensional regressors in the two surveys
[*] p=0.001
[**] p=0.001
TTO time trade off, *VAS* visual analogue scale, *SRH* self-rated health

Supplementary Table S9 Estimated TTO and VAS values for SRH

Self-Rated Health	Estimated TTO value	Estimated VAS value
Very good health	0.9751	92.16
Good health	0.9442	83.36
Fair health	0.8349	65.63
Poor health	0.6585	41.64
Very poor health	0.4959	28.05

TTO time trade off, *VAS* visual analogue scale, *SRH* self-rated health

Supplementary Table S10 Regression analysis, comparison of TTO and VAS values, SRH, main effect model, pooled data

	TTO		VAS	
	Model 1 SRH		Model 1 SRH	
**Variable**	**Estimate**	**p-value**	**Estimate**	**p-value**
**Intercept**	0.9768	<0.0001	92.21	<0.0001
**SRH** [a]				
Good health	-0.0296	<0.0001	-8.76	<0.0001
Fair health	-0.1394	<0.0001	-26.17	<0.0001
Poor health	-0.3311	<0.0001	-50.55	<0.0001
Very poor health	-0.4939	<0.0001	-64.50	<0.0001
**Observations**	38,941		38,941	
**Adjusted R^2**	0.2417		0.5235	

[a] Reference group: very good SRH
TTO time trade off, *VAS* visual analogue scale, *SRH* self-rated health

Supplementary Table S11 Regression analysis on TTO and VAS values, SRH, main effect model, comparison by survey

	TTO					VAS			
	Model 1 SRH					Model 1 SRH			
	2004 survey		2006 survey			2004 survey		2006 survey	
**Variable**	**Estimate**	**p-value**	**Estimate**	**p-value**		**Estimate**	**p-value**	**Estimate**	**p-value**
**Intercept**	0.9670	<0.0001	0.9804	<0.0001		91.60	<0.0001	92.68	<0.0001
**SRH** [a]									
Good health	-0.0322	<0.0001	-0.0292	<0.0001		-8.05	<0.0001	-9.49	<0.0001
Fair health	-0.1391	<0.0001	-0.1399	<0.0001		-25.94	<0.0001	-27.07	<0.0001
Poor health	-0.3134	<0.0001	-0.3189	<0.0001		-51.78	<0.0001	-48.93	<0.0001
Very poor health	-0.4670	<0.0001	-0.4949	<0.0001		-63.52	<0.0001	-64.66	<0.0001
**Observations**	23,548		21,188			19,785		21,339	
**Adjusted R^2**	0.2200		0.2304			0.5330		0.5286	

[a] Reference group: very good SRH
TTO time trade off, *VAS* visual analogue scale, *SRH* self-rated health

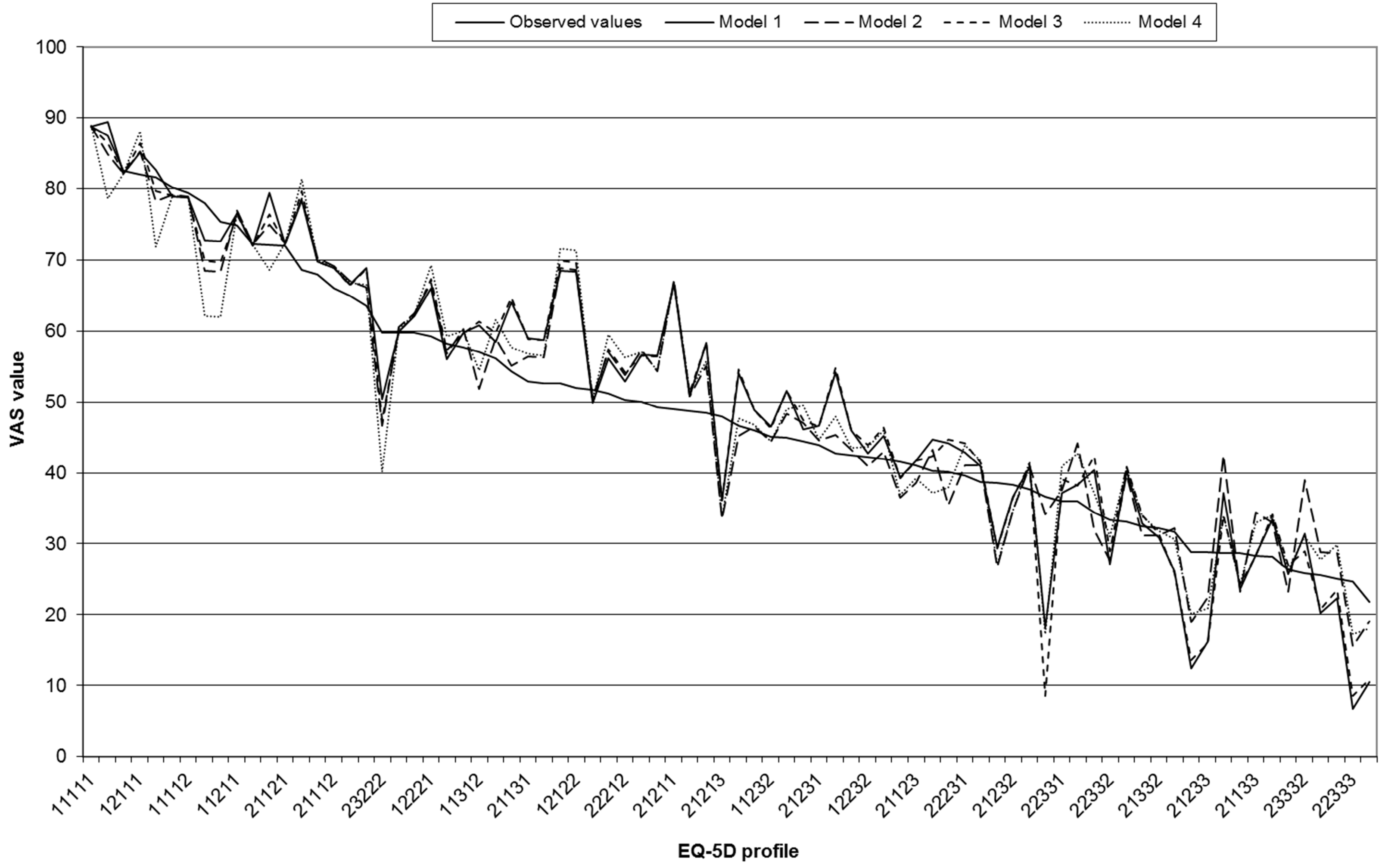

Supplementary Fig. S1 Estimated VAS values predicted by different OLS models compared to the observed mean VAS values for health states with five or more observations (n=80)
